# Molecular evidence of the absence of *Metagonimus yokogawai* (Katsurada, 1912) in Europe: report of *Metagonimus* sp. in cyprinoid fish from the River Danube in Hungary

**DOI:** 10.1007/s00436-023-07932-1

**Published:** 2023-08-12

**Authors:** Gábor Cech, Martina Gyöngy, Diána Sándor, Kálmán Molnár, Boglárka Sellyei, Ádám Varga, Csaba Székely

**Affiliations:** 1 Veterinary Medical Research Institute, Budapest, Hungary; 2 University of Debrecen, Juhász-Nagy Pál Doctoral School, Department of Hydrobiology, Debrecen, Hungary; 3 Eötvös Loránd University, Doctoral School of Biology Program of Zootaxonomy, Animal Ecology and Hydrobiology, Budapest, Hungary

**Keywords:** *Metagonimus*, Digenean trematodes, Metacercariae, Freshwater fish

## Abstract

Trematodes of the genus *Metagonimus* Katsurada, 1912 (Digenea: Heterophyidae) are zoonotic parasites that cause infections in humans, with most cases reported in Southeast Asia. Larvae from the second intermediate host, called metacercariae, of one of human-infecting species, *M. yokogawai* (Katsurada, 1912), have been reported from cyprinoid fish in Europe. In the present study, we provided DNA-based evidence that metacercariae of *Metagonimus*, which are commonly found in the scales of various cyprinoids in Central Europe (Danube River in Hungary) do not belong to *M. yokogawai*. Sequence analysis of the ITS region, 28S rDNA, and cox1 genes showed that this species is clearly distinct from all Asian species, including *M. yokogawai*, which probably does not occur in Europe. Metacercariae from cyprinoids might belong to *Metagonimus romanicus* (Ciurea, 1915), an insufficiently known species described from Romania.

## Introduction

Trematodes of the family Heterophyidae are common parasites of birds and mammals (Pearson, 2008). Some species of the so-called small intestinal flukes are causative agents of fish-borne diseases in humans, with human cases reported from Southeast Asia (Chai et al. 2005; Chai and Jung 2017). Five species of the heterophyid genus *Metagonimus* Katsurada, 1912 have also been detected in humans (Chai 2015), with most cases caused by *M*. *yokogawai* (Katsurada, 1912), the type species of the genus. This species was described by Katsurada (1912) in Japan as *Heterophyes yokogawai* and then reported from other Asian countries (South Korea, Taiwan, and India), as well as Russia, Israel and several European countries (Bulgaria, Czech Republic, Hungary, Serbia, and Spain) (Yu and Mott 1994; Rácz and Zemankovics 2002; Chai et al. 2009; Pornruseetairatn et al. 2016). However, the global distribution of *M. yokogawai* has yet to be confirmed, as other species of *Metagonimus* have a limited geographical distribution, with most species described from East Asia (Shimazu 1999, 2002; Chai and Lee 2002; Shimazu and Urabe 2002; Kino et al. 2006; Shumenko et al. 2017; Tatonova et al. 2018; Nakao et al. 2022).

*Metagonimus* metacercariae have been reported from freshwater fish in eastern and central Europe, especially from the Danube River basin (Vojtek 1959; Žitňan 1969; Kulišić and Lepojev 1994). In Hungary, Prettenhoffer (1930) reported metacercariae identified as *M. romanicus* (Ciurea 1915), a species described insufficiently by Ciurea (1915) from Romania. In contrast, Molnár (1969), Rácz and Zemankovics (2002), and Molnár and Baska (2017) identified metacercariae found in cyprinoids as *M. yokogawai*.

In the present work, we provide molecular evidence that *Metagonimus* metacercariae commonly found in cyprinoids in Hungary and adults from experimental infections do not belong to *M. yokogawai*, suggesting that this trematode infecting humans is not present in Europe as previously thought.

## Material and methods

### Examination and collection of metacercariae of Metagonimus sp.

In the present study, 131 cyprinoid fish (Cyprinoidei: Leuciscidae) were collected from the Danube River at Zebegény (47°48'N, 18°55'E) and Szentendre (47°40' N, 19°4'E). Between 2015 and 2017, 9 white bream (*Blicca bjoerkna*), 26 freshwater bream (*Abramis brama*), 42 bleak (*Alburnus alburnus*), 4 ide (*Leuciscus idus*), 31 common nase (*Chondrostoma nasus*) and 5 chub (*Squalius cephalus*) were collected. Between 2020 and 2021, only 4 common nase and 6 chub were taken from the Danube, while two chub and two vimba bream (*Vimba vimba*) were caught in 2022. In addition, 19 European perch (*Perca fluviatilis*) were taken from Lake Balaton between 2020 and 2021, one of which was also infected with *Metagonimus* metacercariae (Table 1). The fish were caught with a 15-m seine net and brought to the laboratory alive in oxygenated plastic bags.

**Table 1:** Occurrence of metacercariae of Metagonimus sp. in fish from the Danube and Lake Balaton in Hungary

Fish species (common name)	Year	Locality	Fish infected/examined (prevalence)
*Abramis brama* (common bream)	2015–2017	River Danube	26/26 (100%)
*Alburnus alburnus* (bleak)	2015–2017	River Danube	42/42 (100%)
*Blicca bjoerkna* (white bream)	2015–2017	River Danube	9/9 (100%)
*Chondrostoma nasus* (common nase)	2015–2017	River Danube	31/31 (100%)
	2020–2021	River Danube	2/4 (50%)
*Perca fluviatilis* (perch)	2020–2021	Lake Balaton	1/19 (5%)
*Squalius cephalus* (chub)	2015–2017	River Danube	5/5 (100%)
	2020–2021	River Danube	2/6 (33%)
	2022	River Danube	2/2 (100%)
*Vimba vimba* (vimba bream)	2022	River Danube	2/2 (100%)
			126/150 (84.0%)

Fish were anaesthetized by clove oil and then decapitated. The body surface and scales were examined under a dissecting microscope. Metacercariae were isolated with a fine needle or by tissue digesting in 0.5% pepsin solution (2 l tap water, 10 g pepsin based on 1: 10,000 NF powder (Molar Chemicals, Halásztelek, Hungary) and 16 ml 25% hydrochloric acid (HCl) at a temperature of 40 °C with stirring. Encysted metacercariae were fixed in 70% ethanol. They were excysted from their capsules using a solution of 50 ml distilled water, 2.5 g pancreatin, and 0.25 g NaHCO_3_ (Fried, 1994). Excystation performed at 27 °C for 5–10 min, and then the metacercariae were placed in 0.9% physiological saline to avoid overdigestion. After treatment, the released metacercariae were observed under the microscope and kept alive for further use, including experimental infections.

### Experimental infection for recovery of adult trematodes

To obtain adults, chicks, ducks, and Syrian hamsters were experimentally infected (permit number PEI/001/1004–4/2015, PEI/001/1792–4/2014) with metacercariae of *Metagonimus* sp. (50 cysts per animal) from cyprinoid fish in 4 experiments (Table 2). Experimental animals were necropsied in accordance with European animal welfare regulations. Adult *Metagonimus* specimens were collected from both the duodenum and faeces of the animals after decantation. The isolated trematodes were considered as adults when they were oviparous. Adult trematodes found were measured and fixed in molecular-grade ethanol for DNA sequencing.

**Table 2:** Experimental results of chicks, ducklings and Syrian hamsters infected with *Metagonimus* metacercariae

Experimental event	Number of animals in the experiment (N)	Number of infected/examined animals (N)	Fish host of the metacercariae	Time interval of the experiment	Results (number of obtained adult specimens)
1	2	2/2 (chicks)	bleak	9 February 2016 – 19 February 2016	5
2	3	3/3 (chicks)	bleak	3 October 2017 – 12 October 2017	13
	1	0/1 (chicks)	chub		negative
	2	2/2 (chicks)	ide		57
3	1	1/1 (ducklings)	common nase	10 May 2019 – 16 May 2019	26
4	2	2/2 (Syrian hamsters)	common nase	11 July 2018 – 20 July 2018	27
Summary	*Metagonimus* sp.: 130 adult specimens				

### Molecular methods

For DNA extraction, samples preserved in 80% ethanol were centrifuged at 8,000 g for 5 min, then the ethanol was removed with a pipette and/or by evaporation with a vacuum centrifuge. DNA was extracted using a Geneaid DNA Mini Kit (Geneaid, Taipei City, Taiwan) and eluted into 100 μl of AE buffer according to the manufacturer’s recommendations. The ITS region (part of 18S rDNA, ITS1, 5.8S rDNA, ITS2, and part of 28S rDNA) was amplified by nested PCR. Primers S18 (5′-TAACAGGTCTGTGATGCC-3′) and L3T (5′-CAACTTTCCCTCACGGTACTTG-3′) (Jousson et al. 1999) were used in the first run. The reaction mixture consisted of 14.4 μl nuclease-free water, 2.5 μl of 10 × DreamTaq buffer (Thermo Scientific, Vilnius, Lithuania), 0.1 μl of DreamTaq Polymerase (1 U; Thermo Scientific), 0.2 mM dNTPs (Thermo Scientific), 0.5 μM of each primer and 2 μl of the extracted DNA in a final volume of 25 μl. The PCR profile consisted of an initial denaturation step at 95 °C for 3 min, followed by 40 cycles at 95 °C for 30 s, 50 °C for 30 s and 72 °C for 2 min, and was terminated with a terminal extension at 72 °C for 5 min and then stored at 4 °C. Primers D1 (5′-AGGAA-TTCCTGGTAAGTGCAA-3′) and D2 (5′-CGTTACTGAGGGAATCCTGGT-3′) (Galazzo et al. 2002) were used in the second run, in 50 μl reaction mixture with the same concentrations as in the first round. 1 μl template form the first PCR round was added to each sample. The second round of PCR consisted of an initial denaturation step at 95 °C for 3 min, followed by 30 cycles at 95 °C for 30 s, 56 °C for 30 s, 72 °C for 2 min, and a final extension step at 72 °C for 5 min, followed by storage at 4 °C. The 28S rRNA gene was amplified using the following primers: DIG12 (5′-AAGCATATCACTAAGCGG-3′), 1500R (5′-GCT ATC CTGAGGGAAACTTCG-3′) according to the protocol of Tkach et al. 2003. The cox1 gene was amplified and sequenced with primers JB3 (5′-TTTTTTGGGCATCCTGAGGTTTAT-3′) and JB4.5 (5′-TAAAGAAAGAACATAATGAAAATG-3′) (Bowles and McManus 1994). The conditions used were identical to those for ribosomal markers, except for the annealing temperature (52 °C).

PCR products were electrophoresed in 1.0% agarose gels in Tris–acetate-EDTA (TAE) buffer gel, stained with 1% ethidium bromide, and then purified using an EZ-10 Spin Column PCR Purification Kit (Bio Basic Inc., Markham, Canada). The purified PCR products from the ITS region, 28S rDNA, and coxI were sequenced with the PCR primers and two additional internal primers 5.8Sr (5′-TGTCGATGAAGAGCGCAGC-3′) and 5.8S2 (5′-TAAGCCGACCCTCGGACAGG-3′) (Tkach et al. 2003) for the ITS region and with 1200R (5′-GCATAGTTCACCATCTTTCGG-3′) (Shumenko et al. 2017) for 28S rDNA. ABI BigDye Terminator v3.1 Cycle Sequencing Kit was used for sequencing, and sequences were read at the MTA SZBK Sequencing Platform in Szeged, Hungary, using an ABI Prism 3100 Genetic Analyser (Thermo Fisher Scientific, Waltham, USA).

### Phylogenetic analysis

Sequence fragments were assembled using MEGA X (Kumar et al. 2018), and ambiguous bases were manually corrected based on the ABI chromatogram. Reference sequences were downloaded from GenBank (see below) with the program BLAST and then aligned with the program MEGA X (Kumar et al. 2018) using the algorithm CLUSTAL W (Thompson et al. 1994). Pairwise distance estimates representing genetic distances were determined using the p-distance model. Phylogenetic analysis was performed using the Maximum Likelihood (ML) method. The best-fitting nucleotide substitution model defined by the Akaike Information Criteria (AIC) was used to analyse the data set. The robustness of the ML phylogenetic tree was determined using bootstrap values based on 1000 resampled datasets. Bayesian Inference (BI) analysis was performed using Geneious Prime® 2019.2.3 software with the MrBayes (Huelsenbeck and Ronquist 2001) plug-in. Posterior probabilities (PP) were estimated over 1,000,000 generations by two independent runs of four simultaneous MCMCMC chains, with every 100th tree saved. The first 25% of the sampled trees were discarded as ‘burn-in’. Phylogenetic trees were visualised using the Tree Explorer of MEGA X.

Sequences of the following species were used for comparison: *M. ciureanus* (AY245702), *M. hakubaensis* (LC576458, LC576462, KM061388–90), *M. katsuradai* (KM061391–93), *M. kinoi* (LC5999533-34, LC666755–56, LC666627–29), *M. kogai* (LC666749–50), *M. miyatai* (LC375946, HQ832633, KM061409–11), *M. otsurui* (KM061394–96, KM061421–23), *M. pusillus* (MF406209–10, MF407172–73), *M. saitoi* (LC666745–46), *M. shimazui* (LC666753-54), *M. suifunensis* (KX387456, KX387520–24, KX387459–60, MK736844, MK736869, MN116490, MN116492), *M. takahashii* (HQ832636), *M. yokogawai* (AB470519, HQ832639, KC330755, KJ631740, KM061412, KX832895, KX857497, OK166789) and *Metagonimus* sp. (LC422948, LC422951).

Sequences of *Metagonimus* sp. were deposited in the GenBank under the accession numbers OQ281688-OQ281703, OQ286093-OQ286097, OQ286071-OQ286088 and OQ308609 (Table 3).

**Table 3:** List of the sequenced metacercariae and adults of *Metagonimus* spp

	Sample	Host	Developmental stage	Date of collection	Site of collection	28S rDNA	ITS	coxI
1	MM2	Rudd (*Scardinius erythrophthalmus*)	Metacercaria	17.02.2016	Danube – Szentendre	OQ286071	OQ286093	OQ281688
2	ME1	Chick (infection 1)	Adult	17.02.2016	experimental infection ETC	OQ286072	OQ286094	OQ281689
3	ME2	Chick (infection 1)	Adult	17.02.2016	infection (09.02.2016)	OQ286073	OQ286095	OQ281690
4	ME3	Chick (infection 1)	Adult	12.10.2017	infection (09.02.2016)	OQ286074	OQ286096	OQ281691
5	ME4	Chick (infection 2)	Adult	12.10.2017	infection (03.10.2017)	OQ286075		OQ281692
6	ME5	Chick (infection 2)	Adult	12.10.2017	infection (03.10.2017)	OQ286076		OQ281693
7	ME6	Chick (infection 2)	Adult	12.10.2017	infection (03.10.2017)	OQ286077		OQ281694
8	ME7	Chick (infection 2)	Adult	12.10.2017	infection (03.10.2017)	OQ286078		OQ281695
9	ME8	Chick (infection 2)	Adult	12.10.2017	infection (03.10.2017)	OQ286079		OQ281696
10	ME9	Chick (infection 2)	Adult	12.10.2017	infection (03.10.2017)	OQ286080		OQ281697
11	SPM2	Perch (*Perca fluviatilis*)	Metacercaria	20.11.2020	Lake Balaton – Balatonszemes	OQ286088		
12	D3UM	Chub (*Squalius cephalus*)	Metacercaria	09.07.2021	Danube – Szentendre	OQ286081		OQ281698
13	D3PM	Chub (*Squalius cephalus*)	Metacercaria	09.07.2021	Danube – Szentendre	OQ286082		OQ281699
14	P3PM	Nase (*Chondrostoma nasus*)	Metacercaria	13.07.2021	Danube – Szentendre	OQ286086		OQ281702
15	P3FF	Nase (*Chondrostoma nasus*)	metacercaria	13.07.2021	Danube – Szentendre			OQ308609
16	D4PM	Chub (*Squalius cephalus*)	metacercaria	12.07.2021	Danube – Szentendre	OQ286083		
17	P4PM	Nase (*Chondrostoma nasus*)	metacercaria	13.07.2021	Danube – Szentendre	OQ286087		OQ281703
18	D5PM	Chub (*Squalius cephalus*)	metacercaria	13.07.2021	Danube – Szentendre			OQ281700
19	DPM6	Chub (*Squalius cephalus*)	metacercaria	15.06.2022	Danube	OQ286084	OQ28609	
20	DPM7	Chub (*Squalius cephalus*)	metacercaria	15.06.2022	Danube	OQ286085		OQ281701

## Results

### Occurrence of metacercariae of Metagonimus sp. in fish

**Fig. 1:**
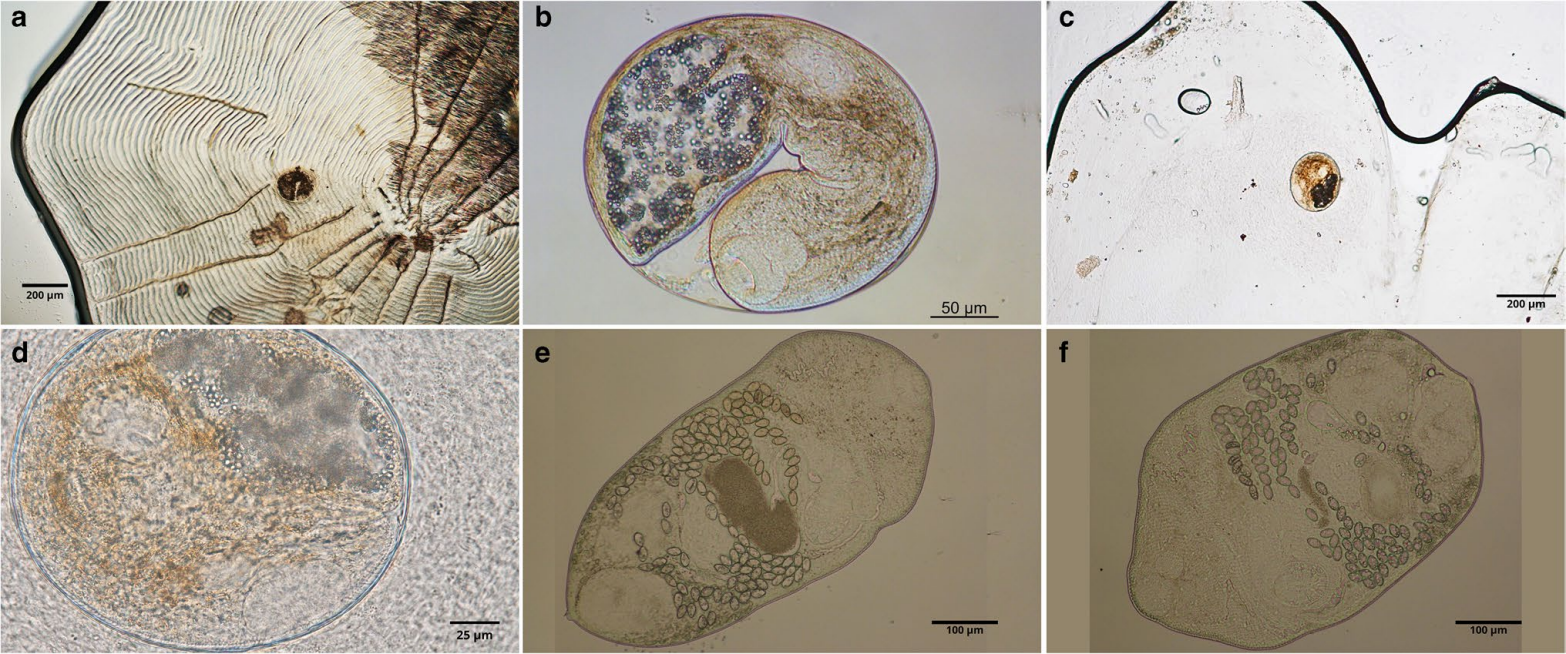
**a**: *Metagonimus* sp. metacercaria embedded in the scales of chub (*Squalius cephalus*) **b**: released after artificial digestion **c**, *Metagonimus* sp. metacercaria in the scales of perch (*Perca fluviatilis*) **d**: and close up **e**: *Metagonimus* sp. adult recovered from a Syrian hamster experimentally infected with metacercariae in this study in ventral view **f**: dorsal view

Metacercariae of *Metagonimus* sp. were found only in the scales of 121 cyprinoid fish of the following species: *Abramis brama*, *Alburnus alburnus*, *Blicca bjoerkna*, *Chondrostoma nasus*, *Leuciscus idus, Squalius cephalus*, and *Vimba vimba* from the Danube (see Table 1). In addition, a European perch (*Perca fluviatilis*) from Lake Balaton was also infected with metacercariae. The metacercariae were encysted, located on the inner side of the scales, and the cysts appeared as small, pearly, roundish structures 180–240 µm long and 140–190 µm wide (Fig. 1a-d).

### Morphology of metacercariae of Metagonimus sp.

Cysts (n = 30) round, 215 ± 14 (205–225), or oval 207 ± 23 (180–240) long by 160 ± 21 (214–190) wide. Encysted metacercariae with clearly visible oral sucker, pharynx, and excretory vesicle. Excysted metacercariae (n = 15) 471 ± 34 (427–507) long, 240 ± 32 (196–280) wide. Body covered with numerous spines. Oral sucker spherical, 67 ± 3.1 (63–70) in diameter. Prepharynx short; pharynx somewhat wider than longer, 33 ± 2 (31–35) long and 37 ± 2 (35–39) wide; oesophagus short, 72 ± 2 (70–74) long. Intestinal caeca narrow, long, ending at level of testes. Ventral sucker elliptical, 30 ± 5 (26–32) long and 28 ± 5 (24–30) wide. Excretory bladder Y-shaped, filled with fine granules, lateral branches 110 ± 5 (105–115) µm long.

Adult trematodes (Fig. 1 e–f) were recovered from experimentally infected chicks, ducks, and Syrian hamsters fed with metacercariae of *Metagonimus* sp. from 4 species of fish (Table 2.). Selected biometrical characteristics of experimentally obtained adults are in Table 4.

**Table 4:** Morphological comparison of *Metagonimus yokogawai* (Shimazu and Kino 2015), M*etagonimus* sp. (N = 15) from this study, and *M. romanicus* (Ciurea 1915)

Morphological characteristic	*Metagonimus yokogawai*	*Metagonimus* sp.	*Metagonimus romanicus*
Body length (µm)	753–1074	750–1100	600–1560
Body width (µm)	314–424	330–550	400–540
Oral sucker (µm)	48–59 × 59–65	36–66 × 55–76	81–110
Pharynx (µm)	35–48 × 36–59	33–48 × 35–59	50–63 × 30–44
Length of oesophagus (µm)	63–119	62–93	86–143
Caecal branches (µm)	–	530–510	–
Ventral sucker (µm)	71–92 × 48–57	60–95 × 45–75	96–114
Anterior/left testis (µm)	111–151 × 68–135	95–175 × 88–143	149–198 × 121–182
Posterior/right testis (µm)	95–175 × 55–127	55–110 × 45–95	161–224 × 132–187
Ovaries (µm)	68–103 × 48–79	68–128 × 48–92	830 –145 × 880–165
Seminal vesicle	111–175 × 35–71	180–205 × 77–88	‒
Seminal receptacle	111–175 × 95–159	133–148 × 50–62	‒

### Molecular characterisation of metacercariae and adults of Metagonimus sp.

For the 28S rRNA gene, the final alignment consisted of 782 bp with 657 conserved and 125 variable positions. Amplification of the ITS region yielded products of approximately 1,400 bp long. The alignment of ITS was 1,132 bp long and contained 801 conservative and 328 variable positions. The final alignment of cox1 sequences was 347 bp long and consisted of 186 conservative and 157 variable positions.

**Fig. 2:**
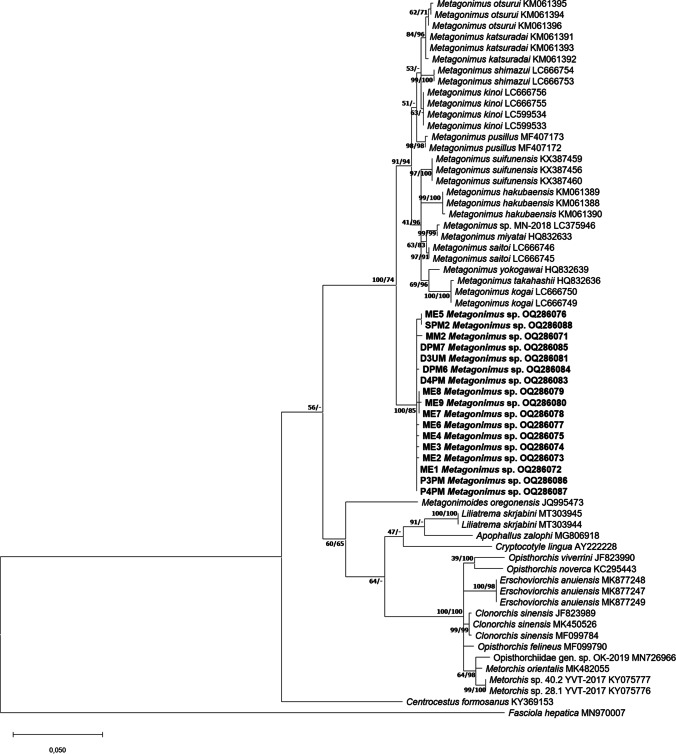

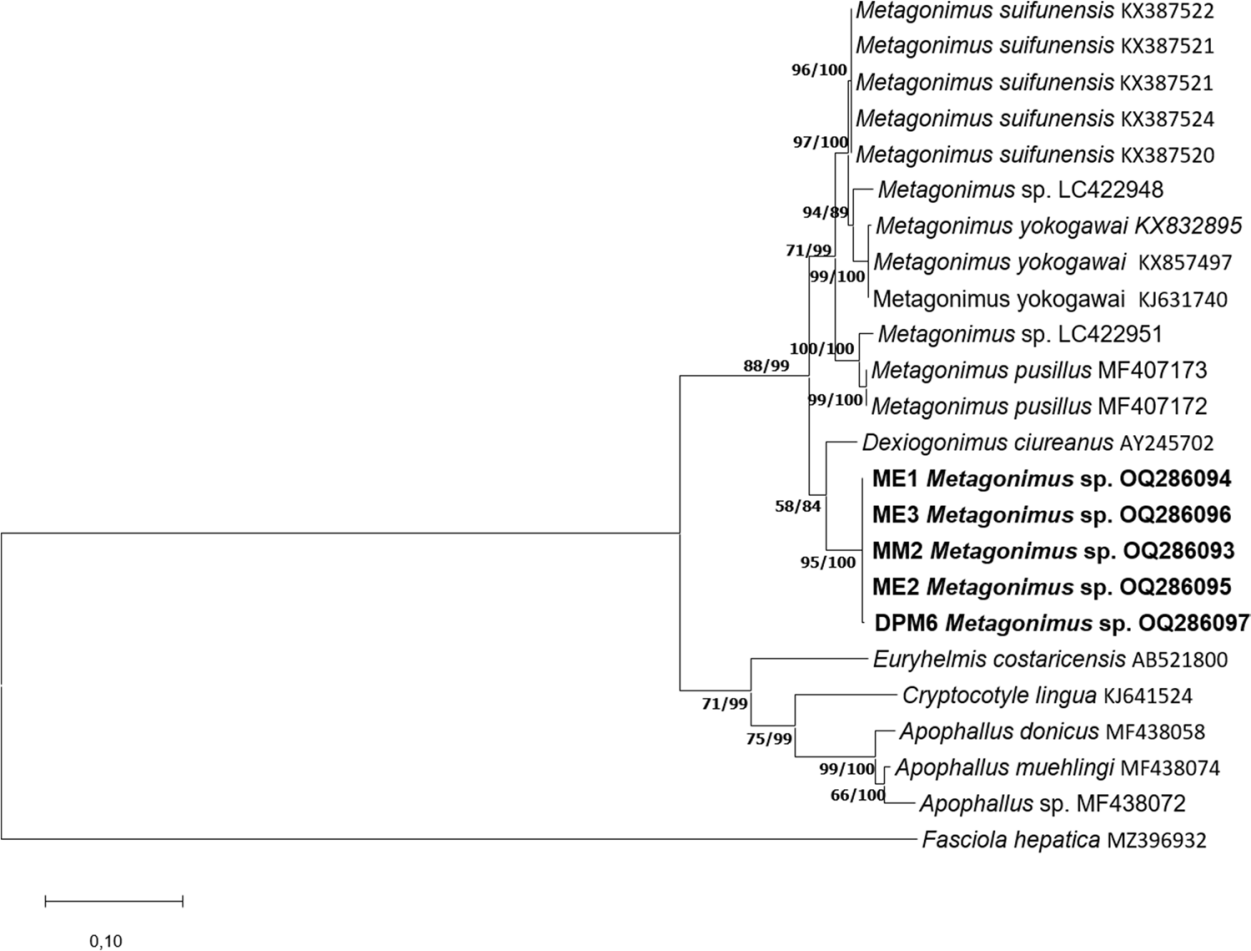

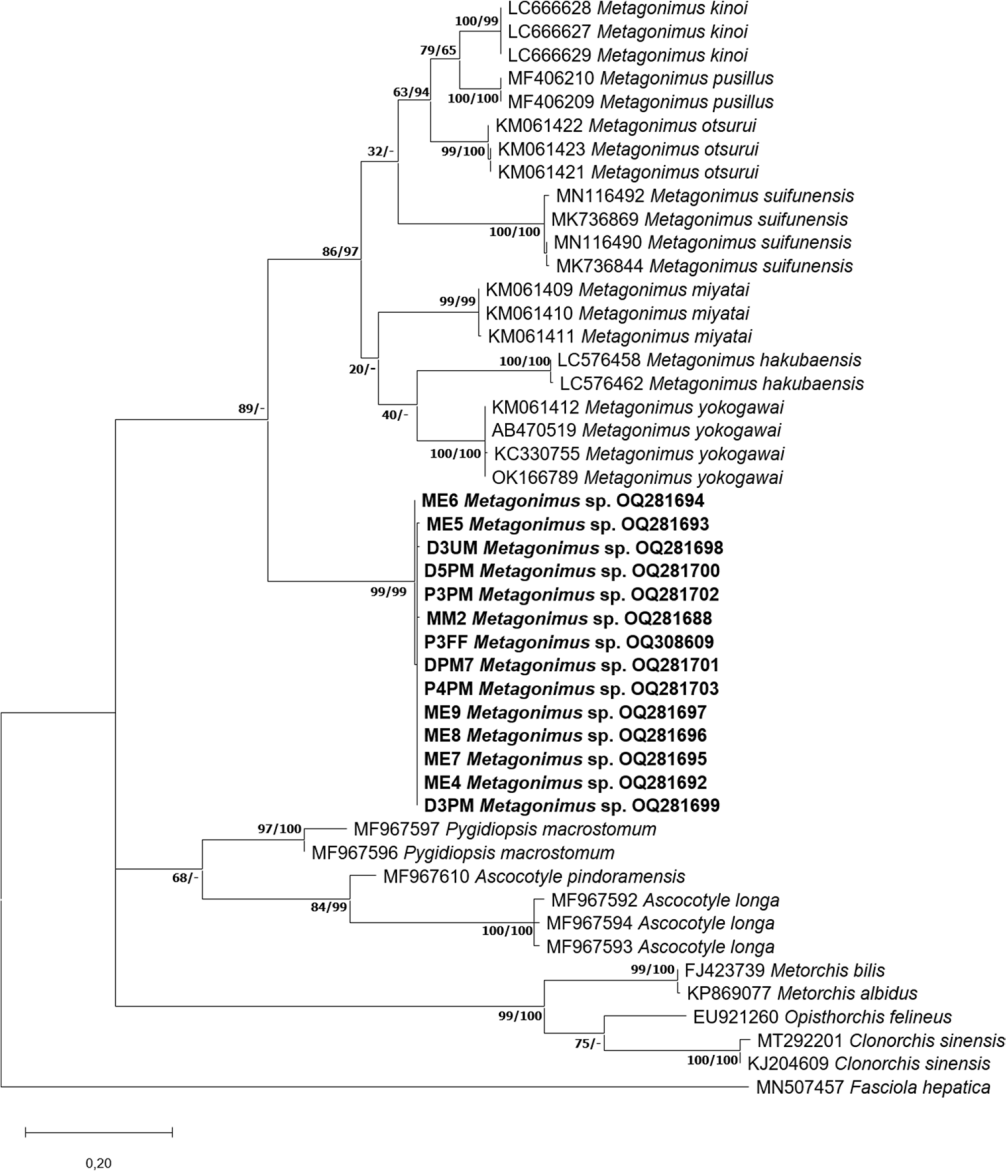
Maximum likelihood tree of the samples of *Metagonimus* spp. from the present study (**a**: 28S rDNA, **b**: ITS region, **c**: coxI) in relation to other heterophyid and opisthorchiid sequences deposited in GenBank. Bootstrap values are indicated at the nodes; posterior probabilities for Bayesian inference are indicated after the bootstrap values. Samples from the present study are in bold. The scale bar indicates the expected number of substitutions per site

The ML and BI phylogenetic analysis of the ITS region, 28S rDNA and cox1 genes revealed that the samples from Hungary formed a single clade distinct from other species of *Metagonimus*, all from East Asia and Far East (Fig. 2).

Results from the 28S rDNA gene also showed that the sequences from metacercariae and the sequences of the adult specimens of *Metagonimus* sp. formed a clade distinct from other species of *Metagonimus*. The clade was located in a basal position in relation to the Asian species. It should be noted that a single metacercaria from European perch was also placed into the same clade as other isolates from the Danube. Within-group patterns of *Metagonimus* sp. were almost identical (mean distance within groups = 0.001), which was also true for the reference sequences of *Metagonimus* species from Asia. The genetic distance (the mean distance between groups) between our samples and Asian *Metagonimus* samples varied between 1.9 and 3.5%, including *M. yokogawai* (2.6%); the difference between individual East Asian species was smaller, ranging from 0.2% to 2.7%.

In the phylogenetic analysis of the ITS region, metacercariae isolated from the Danube (MM2, DPM6) and three adult specimens isolated from experiments (ME1, ME2, ME3) formed a distinct clade. Similarly to the 28S rDNA analysis, *Metagonimus* sp. took a basal position within a monophyletic clade containing all analyses species of *Metagonimus*. Sequences of all isolates of *Metagonimus* sp. from the Danube were identical. *Metagonimus* sp. was most closely related in its 28S rDNA sequences with *Metagonimus ciureanus* (Witenberg 1929) (KX387520), a species of uncertain taxonomic status, which was originally described as *Dexiogonimus ciureanus* in Israel.

The analysis of mitochondrial cox1 gene corresponded to the results of analyses of sequences of nuclear markers. *Metagonimus* sp. samples formed a most basal clade of the monophyletic *Metagonimus*, distinct from other *Metagonimus* species. Mean distance of isolates of *Metagonimus* sp. was only 0.001. Genetic distances between these samples and other *Metagonimus* species ranged from 20.5% to 27.3%. The present specimens were most closely related to *Metagonimus kinoi* (LC666627–29), but similarity was only 20.5% (similarity with *M. yokogawai* was 23.5%). The previously described Asian *Metagonimus* species showed 10.0–25.5% interspecific distances to each other.

## Discussion

In the present study, as many as seven species of cyprinoid fish from the Danube (*Blicca bjoerkna*, *Abramis brama*, *Alburnus alburnus*, *Chondrostoma nasus*, *Leuciscus idus*, *Squalius cephalus* and *Vimba vimba*) were heavily infected with *Metagonimus* metacercariae. Earlier, metacercariae most likely conspecific with those found recently in the Danube fish were identified as *M. romanicus* by Prettenhoffer (1930) and *M. yokogawai* by the other authors, such as Žitňan (1969) from Slovakia, Francová et al. (2011) from the Czech Republic, Cakić et al. (2007) and Djikanovic et al. (2011) from Serbia, Nachev and Sures (2009) and Ondračková et al. (2012) as *M. yokogawai* from Bulgaria.

Genotyping of these metacercariae and adults from experimentally infected definitive hosts showed that they are conspicuously distinct all East Asian species analysed, including *M. yokogawai*. However, the identification of trematodes from Hungary to species level was not possible due to poor quality of adults obtained (specimens were not properly fixed and thus are not suitable for a reliable morphological and biometrical comparison). In addition, comparison of selected metrical data of *Metagonimus* sp. from Hungary and *M. romanicus* has revealed some differences, especially in the size of the oral sucker and pharynx (Table 4). Therefore, specimens from Hungary are tentatively identified as *Metagonimus* sp.

The occurrence of *Metagonimus* sp. in European perch from Lake Balaton is noteworthy because there were only two previous reports of metacercariae of *Metagonimus* in percids in Europe: Cojocaru (2006) found two infected perch in Romania and Bykhovskaya-Pavlovskaya (1962) reported one infected perch in the U.S.S.R.. However, perch is probably only incidental host because prevalence of its infection in Hungary was low and only a single perch was infected with a single metacercaria.

It is possible that *Metagonimus* sp. from the Danube in Hungary is conspecific with *M. romanicus*, which was found in fish from the same river basin in Romania (Ciurea 1915, 1933). However, there are no molecular data on *M. romanicus*, and the available material of *Metagonimus* sp., including adults from experimentally infected hamsters and birds, does not allow us reliable comparison of the two species. In fact, the present study was focused on genotyping metacercariae and adults and insufficient attention was paid to proper processing of trematodes from experimentally infected hosts. In addition, there are some metrical differences between *M. romanicus* and *Metagonimus* sp., especially the size of the oral sucker and pharynx (Table 4). Therefore, it is necessary to obtain properly fixed material of *Metagonimus* sp. and compare it with specimens from Romania, provided they are available. However, there is no doubt that *Metagonimus* sp. from Hungary and *M. yokogawai* are different species because of nucleotide difference between our samples and *M. yokogawai* (ITS: 6.4–6.5%; 28S rRNA: 2.0–3.4%, cox1: 21–22%).

In addition to the genetic differences between *Metagonimus* sp. from Hungary and the Asian species of the genus, they also differ significantly in the site of infection of the metacercariae. Those of *Metagonimus* sp. are localised exclusively in the scales, whereas the metacercariae of all Asian species can be found in the muscles, intestines, gills and scales as well (Kino et al. 2006; Nakao et al. 2022). This site of infection of European specimens makes them less important from an epidemiological point of view, as whole fish with scales are rarely consumed in Europe. In contrast, successful experimental infections of hamsters with *Metagonimus* sp. provide evidence that this species can mature in mammals. Therefore, the potential risk of zoonotic infection with *Metagonimus* sp. from consumption of raw and undercooked fish from the Danube River cannot be ruled out entirely.

## Conclusions

Metacarcariae of the genus *Metagonimus* were found in large numbers in the scales of cyprinoid fishes from the Hungarian Danube. Sequence data from three different loci (28S rDNA, ITS region and cox1) show that they do not belong to the East Asian *M. yokagawai*, which is very unlikely to occur in Europe despite previous records in the literature. The species found in Hungary may be conspecific with insufficiently known *M. romanicus* described by Ciurea (1915) in Romania. Successful infections of hamsters with metacercariae from fish demonstrate the zoonotic potential of this species.

## Data Availability

The sequence data generated during the current study are available in the GenBank repository under the accession numbers OQ281688-OQ281703, OQ286093-OQ286097, OQ286071-OQ286088 and OQ308609.
